# Far away from home? Ancient DNA shows the presence of bicolored shrew (*Crocidura leucodon*) in Bronze Age Denmark

**DOI:** 10.1002/ece3.11680

**Published:** 2024-07-02

**Authors:** Mahsa Mousavi‐Derazmahalleh, Niels Haue, Marie Kanstrup, Jørgen T. Laursen, Sherralee S. Lukehurst, Jacob Kveiborg, Morten E. Allentoft

**Affiliations:** ^1^ Trace and Environmental DNA (TrEnD) Laboratory, School of Molecular and Life Sciences Curtin University Bentley Western Australia Australia; ^2^ North Jutland Museums, Department of Archaeology Frederikshavn Denmark; ^3^ Aarhus AMS Centre, Department of Physics and Astronomy Aarhus University Aarhus Denmark; ^4^ JTL Fauna Consult Brabrand Denmark; ^5^ Department of Archaeological Science and Conservation, Moesgaard Museum Højbjerg Denmark; ^6^ Lundbeck Foundation GeoGenetics Centre Globe Institute, University of Copenhagen Copenhagen Denmark

**Keywords:** bicolored shrew, Bronze Age Denmark, mitochondrial genome, white‐toothed shrews

## Abstract

An excavation of an Early Iron Age village near Aalborg in Denmark uncovered the jaws and skull fragments from a small mammal that were morphologically identified to the genus *Crocidura* (white‐toothed shrews). Three *Crocidura* species are known from prehistoric continental Europe but none of them are distributed in Scandinavia, which is why this surprising finding warranted further analyses. The bone was radiocarbon‐dated to 2840–2750 calibrated years before present (cal. BP), corresponding to the Late Bronze Age and hence earlier than the Iron Age archeological context in which it was found. Using highly optimized ancient DNA protocols, we extracted DNA from one tooth and shotgun‐sequenced the sample to reconstruct a near‐complete mitochondrial reference genome (17,317 bp, 32.6× coverage). Phylogenetic analyses determined this specimen as a bicolored shrew (*Crocidura leucodon*) but with a phylogenetic position basal to the clade of known sequences from this species. The confirmation of *Crocidura* presence in Denmark by the Late Bronze Age sheds new light on the prehistoric natural history of Scandinavia. We discuss the implications of this finding from both zoo‐archeological and ecological perspectives. Furthermore, the mitochondrial genome reconstructed in this study offers a valuable resource for future research exploring the genetic makeup and evolutionary history of Eurasian shrew populations.

## Graphical abstract

This ancient DNA study is the first to confirm the presence of *Crocidura leucodon* in Denmark, and the first recorded presence of any member of the genus in this region in prehistoric times.

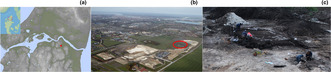

## 1 INTRODUCTION

**FIGURE 1:**
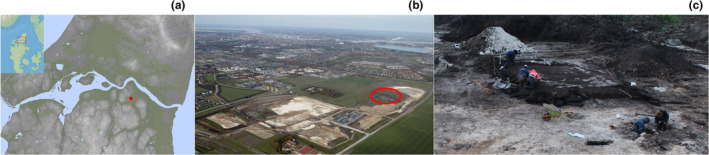
(a) Map of Limfjord Area, Denmark, elevation model using LiDAR. Meadows and wetlands marked in green. The find spot is marked with a red dot. (b) The south‐eastern part of Aalborg with the excavation area marked in red. The Limfjord, dividing Vendsyssel from the rest of Jutland can be seen at the top of the photo. © Region Nordjylland 2018. (c) Excavation of the small building, A14522, with a partly preserved lime floor exposed. The *Crocidura* bones were recovered from wet sieved soil samples within the building. © North Jutland Museums.

Between 2013 and 2021, a large‐scale excavation in the south‐eastern outskirts of Aalborg, Denmark (Figure 1), resulted in a fully excavated rural village from the Early Iron Age. Archeological evidence suggest that the village was established in the Early Pre‐Roman Iron Age (approx. 250 bc), increasing in size to 13 or 14 farms within a few generations and then being abandoned again at the transition between the Early and Late Roman Iron Ages (approx. 175 ad) (Internal report, Danish national database of archeological surveys). The settlement was surrounded by vast meadows to the north and east, and recent studies of the prehistoric coastline indicate that the houses were placed close to the ancient shore, where it is now located more than 5 km from the Limfjord (Kristiansen et al., 2021).

Zoological remains were also recovered from the site, including c. 600 bones from micromammals (Table S1). One particularly unexpected zoological discovery was the identification of two jawbones and skull fragments from the white‐toothed shrew genus (*Crocidura*) of which there are no confirmed recordings from Denmark. The exact species could not be determined confidently from morphological analyses, but it was evident that this was a highly unusual specimen. In addition to filling important knowledge gaps on prehistoric Scandinavian biodiversity, this discovery may hold implications for understanding biodiversity responses to a changing environment. It is widely recognized that small mammals serve as crucial indicator species for monitoring environmental changes, particularly climatic conditions, over time (Hope et al., 2017; Rowe & Terry, 2014).

Conversely, analyses of animal bones that are found severely “out of place” can be key to understanding why some bone assemblages do not accurately reflect the local fauna composition. Such mechanisms could, for example, involve rare long‐range dispersals (Moll et al., 2021; Mukhacheva & Tolkachev, 2022), biased fossil deposition (Allentoft et al., 2010; Kattel et al., 2007), or human‐ or animal‐mediated movement of certain species or their remains (Brace et al., 2016; Bullock et al., 2018). By recognizing the importance and uniqueness of the identified *Crocidura* specimens, we conducted a series of analyses to identify their age and taxonomy.

Shrews are members of the family Soricidae and constitute a group of small mammals that mainly feed on insects and other small invertebrates. Shrews show a high sensitivity to harsh environmental conditions, particularly during the winter season. This is due to their rapid metabolism, small body size, and limited capacity to store energy in the form of fat reserves (Dokulilová et al., 2023). The Soricidae family encompasses three subfamilies including Crocidurinae (white‐toothed shrews), Soricinae (red‐toothed shrews) and Myosoricinae. Myosoricinae is confined to Africa and represents <1% of the all known shrew species but the other two subfamilies have a diverse assemblage of species distributed across the globe from forests to grasslands and wetlands (Neves et al., 2019; Von Merten & Siemers, 2012).

Europe is home to a large diversity of shrew species from the genera *Neomys*, *Sorex* (red‐toothed shrews) and *Crocidura* (white‐toothed shrews). The genus *Neomys* encompasses the water shrews, which are known for their semi‐aquatic lifestyle and adaptations to life near freshwater habitats. Key morphological characteristics of these shrews include larger hind feet fringed with stiff hairs, which extend to their toes, fingers and tail. These fringes serve to increase surface area, improve propellant power, and enhance stability during swimming (Krystufek et al., 2000). The genus *Sorex* is typically terrestrial (Churchfield & Rychlik, 2006), and is broadly distributed in Eurasia and North America (Mackiewicz et al., 2017). Despite similar diet, there are significant mandible muscular and skeletal morphology variations within this genus (Young et al., 2010). The *Crocidura* genus not only differs from its red‐toothed family members in morphology and tooth formula but also lacks pigmentation in its teeth, hence earning its common name, i.e., the white‐toothed shrews. They are the most species‐rich genus among all mammals, currently comprising more than 200 recognized species, many of which are difficult to differentiate solely based on morphological characters (Li et al., 2023). Therefore, additional analyses, such as biochemical, cytogenetic and molecular tests are often necessary to obtain a secure species identification (Rofes & Cuenca‐Bescos, 2011).

In Europe, the three most widespread species of *Crocidura* include the lesser white‐toothed shrew (*Crocidura suaveolens*), the bicolored white‐toothed shrew (*Crocidura leucodon*), and the greater white‐toothed shrew (*Crocidura russula*) (Neves et al., 2021). However, despite their widespread distribution in Europe, in Denmark, shrews from the genus *Crocidura* have been recorded only once in the remains of a barn owl pellet (Laursen, 2024), and never reported from prehistoric material. The species of shrew found in Denmark today include Common shrew, also known as Eurasian shrew (*Sorex araneus*), Eurasian pygmy shrew (*Sorex minutus*), and Eurasian water shrew (*Neomys fodiens*) (Arter.dk, 2023).

Considering the significance of discovering prehistoric *Crocidura* remains in Scandinavia, our study employs a variety of analyses to: (i) determine its chronological age, (ii) obtain a secure species identification, and (iii) establish its phylogenetic position in the context of other shrews.

## 2 MATERIALS AND METHODS

### 2.1 Archeological context and sample description

The skull and jawbone fragments of the *Crocidura* species (specimen's museum number: ÅHM 6023 ×5543) was found within a small building, A14522, in a waste layer of bones and sherds upon a lime floor (Figure 1). The floor measures 4 × 4 m and was surrounded by stones to the north and a large intact pavement to the west. The pavement west of A14522 represents the southern entrance area of longhouse A15624, with a stable in the eastern part, a living area in the western part, and two opposing doors in the middle of the longhouse. The farm thereby consists of a 16‐m‐long longhouse, a well‐preserved stone‐paved entrance area and a smaller outbuilding placed to the south of the stable. The bone fragments emerged during wet sieving of the waste layer using net sizes of 5 mm and 2 mm (applied to 90% and 10% of the samples, respectively). Initial taxonomic identification of the remains was based on dental formula, bone and tooth morphology (Hutterer, 2005; Niethammer & Krapp, 1990; Richter, 1964; Vogel et al., 1989).

### 2.2 Radiocarbon age determination

The radiocarbon age determination was conducted at the Aarhus AMS Centre. The collagen extraction procedure followed a modified version of the widely used Longin Method (Brown et al., 1988; Longin, 1971). A more detailed description is available in Kveiborg and Olsen (2023). Due to the very small sample size (20.59 mg) of the jawbone we extracted collagen without using NaOH and Ultrafiltration (UF), since especially the latter reduces the collagen yield considerably (Jørkov et al., 2006). After collagen extraction, we ended up with 0.46 mg of freeze‐dried collagen, which we transferred to quartz tubes with 200 mg pre‐cleaned CuO and subsequently evacuated, sealed and combusted to CO_2_ at 900°C. Graphitization followed the H_2_ reduction method using an iron catalyst and MgClO_4_ to trap excess water (Santos et al., 2007; Vogel et al., 1984). A very small amount of graphitized Carbon (0.14 mg) was mounted and the measurement took place at the Aarhus AMS Centre (AARAMS) using a HVE 1 MV tandetron accelerator AMS system (Olsen et al., 2017). We report the ^14^C age in conventional radiocarbon years BP (before present = 1950) in accordance with international convention (Stuiver & Polach, 1977).

To ensure a sufficient amount of carbon for the radiocarbon age determination all the collagen was used, which left no collagen for isotope analysis. The species is well known to be 100% terrestrial, so reservoir correction of the date should not be necessary. For calibration of the ^14^C age we use the IntCal 20 (Reimer et al., 2020) and the Oxcal v4.4.4 calibration software (Ramsey, 2009). The probability method calculates the calibrated age ranges corresponding to 68.2% probability (1 σ) and 95.4% probability (2 σ) indicating the probability ranges of the true date.

### 2.3 Ancient DNA extraction, library preparation, and sequencing

All the pre‐PCR work was performed in sterile cleanlab facilities at the Lundbeck Foundation GeoGenetics Centre (Globe Institute, University of Copenhagen). One tooth, a lower (1st) incisor, was removed from the jawbone and crushed. A volume of 2 mL digestion buffer (pr. mL: 929 μL of 0.5 M EDTA, 10 μL of TE buffer, 10 μL of Proteinase K, 50 μL of 10% *N*‐laurylsarcosine, and 1 μL of phenol red) was added to the sample and incubated for 24 h at 42°C. The DNA was then purified using the silica‐in‐solution method similar to Rohland and Hofreiter (2007) but using the optimized binding buffer from Allentoft et al. (2015). The final elution was performed with 50 μL TEB buffer. Double‐stranded blunt‐end libraries were constructed from the extracted DNA using NEBNext DNA Prep Master Mix Set E6070 (New England Biolabs Inc.) with protocol modifications (Allentoft et al., 2015; Orlando et al., 2013), and amplified with double‐indexed Illumina‐specific adapters prepared as Meyer and Kircher (2010). The DNA concentration of the library was quantified on an Agilent 2200 Tapestation, and pooled equimolarly with other libraries (from different projects) before sequenced on an Illumina NovaSeq6000 platform (150 PE) at the Danish National High‐throughput DNA Sequencing Centre. Negative controls were included at both the extraction and library steps, yielding undetectable DNA concentrations.

### 2.4 Bioinformatics

The raw sequencing data were base‐called using the Illumina software CASAVA 1.8.2 and sequences were de‐multiplexed with a requirement of full match of the nucleotide index sequences. The raw sequences were quality checked with FastQC (Andrews, 2010). Adapter sequences and reads with phred quality score below 30 were removed, overlapped paired‐end reads merged, and any merged reads less than 30 bp were discarded using AdapterRemoval2 (Schubert et al., 2016). To preliminarily determine if the specimen was more related to *Sorex*, *Neomys* or the *Crocidura* genus trimmed sequence reads were mapped against multiple mitochondrial reference genomes using BWA aln (Li & Durbin, 2009), with default parameters. The complete list of mitochondrial genomes used for mapping is available in Table 1. Reads with mapping quality lower than 25 and potential PCR duplicates were removed from the alignment by SAMtools. (Li et al., 2009) The assembly of mitogenome sequences was performed using MITObim v1.9.1 (Hahn et al., 2013) in quick mode with mismatch 2, using the following complete mitogenome references as bait: *Crocidura russula*, *Crocidura tanakae*, *Crocidura sibirica, Crocidura. negrina, Crocidura shantungensis*. The accession number of these mitogenomes is provided in Table 1. These references were chosen to cover a varying phylogenetic distance (within the *Crocidura* genus) and based on their availability on GenBank at the time. All the trimmed sequences were then re‐mapped with BWA aln against each of these draft assemblies (allowing n6 and n7 mismatches) and the resulting bam‐files were inspected visually in Geneious Prime 2022.1.1 (https://www.geneious.com). Assemblies that yielded regions with low coverage in this re‐mapping exercise were excluded at this stage. Two assemblies performed well and were highly congruent in the re‐mapping exercise and these were aligned (for both n6 and n7; i.e., a total of four assemblies) to construct a single consensus sequence serving as our reference assembly. As a final check for equal mitogenome‐wide coverage, trimmed sequences were mapped again to this consensus assembly using BWA aln (same parameters as above). Mitogenome annotation was performed on this final assembly using MitoZ (Meng et al., 2019) and the annotations were manually checked and curated in Geneious. To assess the ancient DNA authenticity of the mapped reads, MapDamage2 (Jónsson et al., 2013) was run using the assembly as reference.

**TABLE 1:** List of reference genomes used for mapping and building initial mitogenomes.

Reference mitochondrial genome	Genbank accession	Number of unique mapped reads after duplicates removed (rmdup)
*Neomys fodiens*	KM092492.1	240
*Sorex araneus*	KT210896.1	235
*Crocidura russula H1*	AY769263.1	775
*Crocidura sibirica*	MH349094.1	1035
*Crocidura negrina*	KR537881.1	978
*Crocidura tanakae*	NC_046831.1	935
*Crocidura shantungensis*	JX968507.1	959

### 2.5 Phylogenetics

For the phylogenetic analysis we retrieved from GenBank 23 and 21 complete or near‐complete *Cytb* and *COI* mitochondrial sequences of shrew species with lengths of 1140 and 1545 bp, respectively (Table S2), including the known shrew species from Denmark (*Sorex araneus*, *Sorex minutus*, *Neomys fodiens*). The same regions were extracted from our own consensus sequence and aligned in Geneious Alignment (https://www.geneious.com), using default parameters. jModelTest v2.1.10 (Darriba et al., 2012) was employed to find the best evolutionary model of nucleotide substitution in this alignment.

Two independent phylogenetic analyses were performed using these two genes. We performed a Bayesian Inference (BI) analysis in MrBayes v3.2.6 (Huelsenbeck & Ronquist, 2001) using a GTR invgamma model with nucleotide sites partitioned for 1 million generations sampling every 500 generations and a 100,000 burn‐in length. To reconstruct the maximum‐likelihood phylogeny, we used IQ‐Tree v2.1.2 (Minh et al., 2020) with automatic model selection and 20,000 bootstraps. The resulting files of both phylogeny programs are visualized in FigTree v1.4.4 (Rambaut, 2006).

## 3 RESULTS

### 3.1 Morphological assessment

**FIGURE 2:**
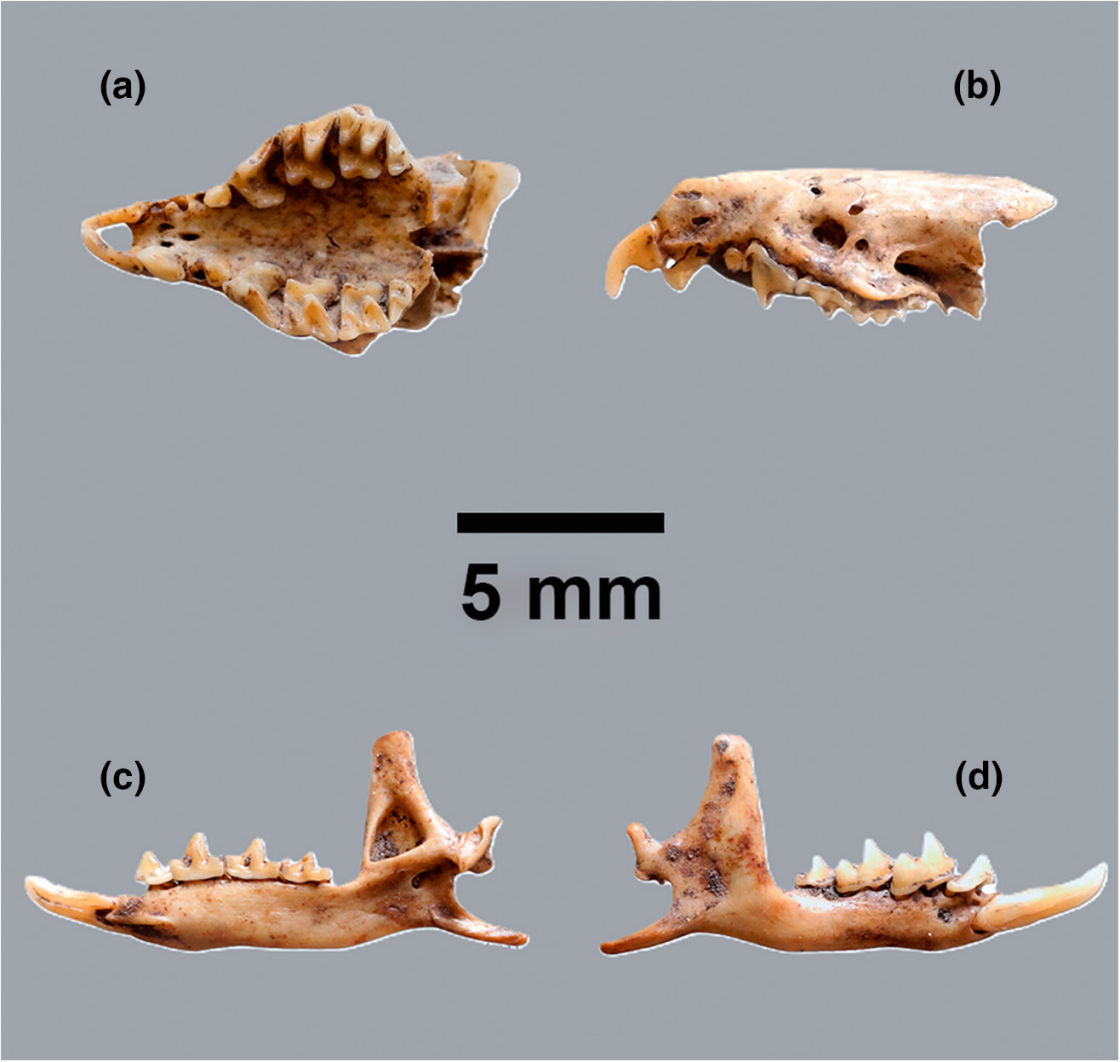
Skull (a + b) and right mandible (c + d) of bicolored shrew, *Crocidura leucodon* from Bronze Age, Denmark. (a) ventral view, (b) lateral view, (c) lingual view, and (d) lateral view. Photographs: Jonas Ogdal Jensen, Moesgaard Museum.

The *Crocidura* findings from this site consist of a partly preserved calvarium and a left and right mandible respectively (Figure 2). The white‐toothed shrews have three upper unicuspid teeth and the dental formula: $\frac{3\;1\;1\;3}{2\;0\;1\;3}$ and are thus easily distinguished from other species of insectivores (Hutterer, 2005). Based on the size, the remains were deemed on morphological grounds to most likely represent *C. leucodon* or *C. russula*, which are of similar size, whereas *C. suaveolens* is rather smaller. Based on specific diagnostic landmarks on the mandible (processus angularis) as described by Niethammer and Krapp (1990), and Richter (1964), an initial identification to *C. russula* was suggested. However, this identification was contradicted by the morphology of the skull especially the shape of the upper P4 and a short rostrum, which points toward *C. leucodon* (Niethammer & Krapp, 1990). Other diagnostic features such as the width of the infraorbital bridge (cf. Vogel et al., 1989) and size of the upper unicuspid teeth (Niethammer & Krapp, 1990) are of intermediate forms or difficult to assess due to fragmentation and loss of teeth (Figure 2). Based on this, the biogeographical range and history of white‐toothed shrews in Northern Europe we sampled one of the mandibles for aDNA analysis and radiocarbon dating.

### 3.2 Radiocarbon dating

The ceramics unearthed in connection with the farm date to the later part of the Pre‐Roman Iron Age. In total, 63 samples (excluding the shrew) from the settlement have been ^14^C‐dated, and all of them date to the period 250 bc–175 ad (Internal report, Danish national database of archeological surveys). However, micromammals are notoriously difficult to date based on context alone in open environments, which is why direct radiocarbon dating of the specimen was crucial.

**FIGURE 3:**
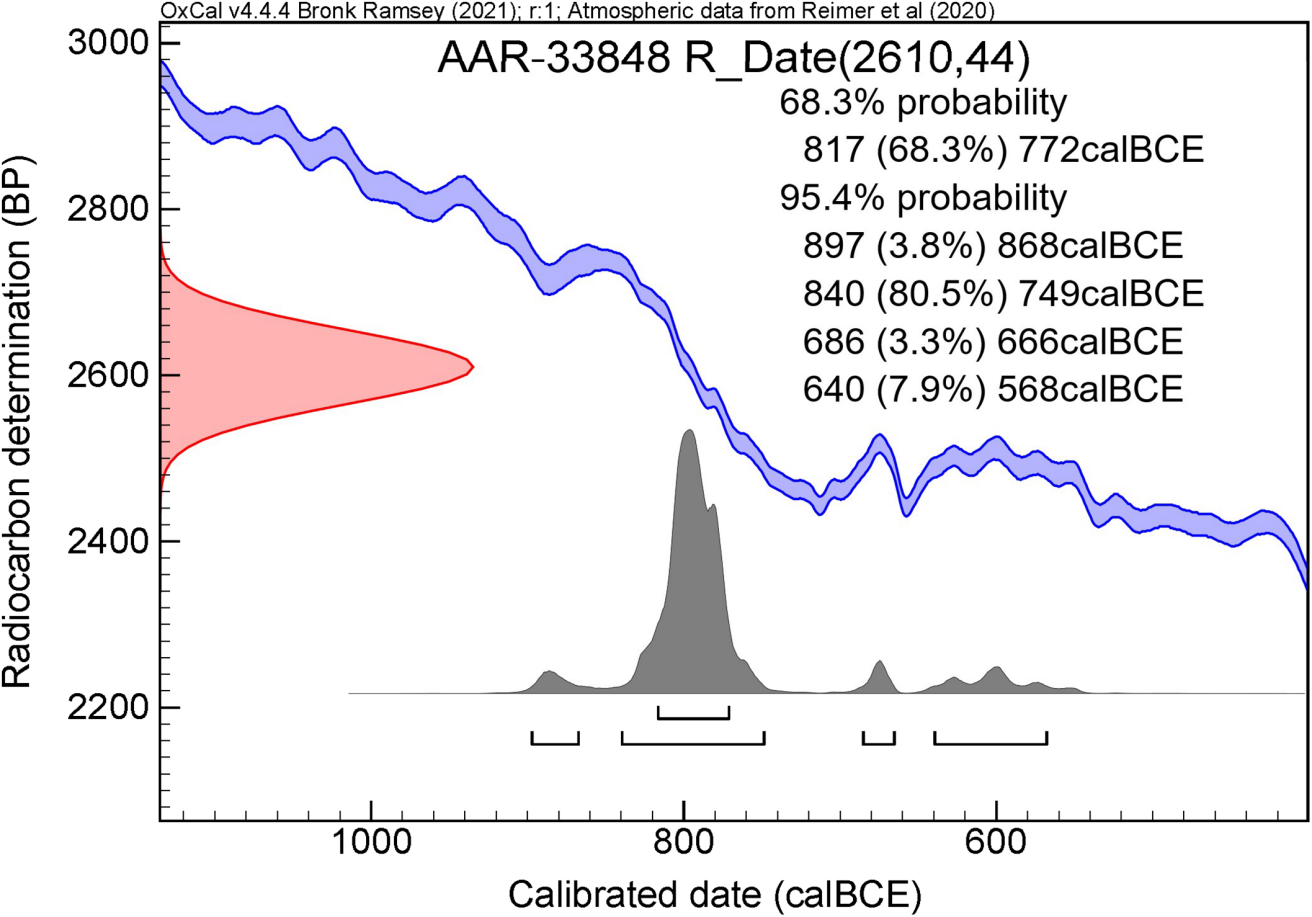
Visualization (single plot option) of the calibrated age of the radiocarbon age determination of the jawbone.

The result of the radiocarbon age determination of the jawbone (sample AAR‐33848) came out as 2610 ± 44 BP. The 95% calibrated probability distribution was 897–568 calBCE but with most of the distribution (80.5%) within 840–749 calBCE. This points toward a date in the Late Bronze Age (Figure 3).

### 3.3 Bioinformatics

We obtained 136,246,905 raw sequencing reads, of which 133,699,835 were retained after quality filtering. These were initially mapped against seven shrew mitochondrial genomes including representatives of the *Crocidura* genus and both the genera known from Denmark (*Neomys* and *Sorex*). The number of uniquely mapped reads (i.e., mapped to only one location on the reference mitochondrial genome) with duplicates removed is presented in Table 1. The *Neomys* and *Sorex* species had lower number of mapped reads (<250) compared with members of the *Crocidura* genus (>775) suggesting that our sample had higher genetic affinity with the latter.

The mitochondrial genome assemblies using five different *Crocidura* sequences as reference baits resulted in draft assemblies with slightly varying sequence length (16,395 to 17,589 bp). When re‐mapping our data against each of these five draft assemblies the visual inspection of the bam‐files revealed multiple low coverage areas within the assemblies that were constructed with baits from *C. sibirica, C. negrina*, and *C. shantungensis*. These three assemblies were therefore not used to construct the final consensus assembly.

The consensus created from the alignment of the two assemblies resulting from using *C. tanakae* and *C. russula* as baits respectively (with 99.5% pairwise identity) had the total length of 17,317 bp. The final average coverage obtained from remapping of sequences to this final consensus was 32.6X.

**FIGURE 4:**
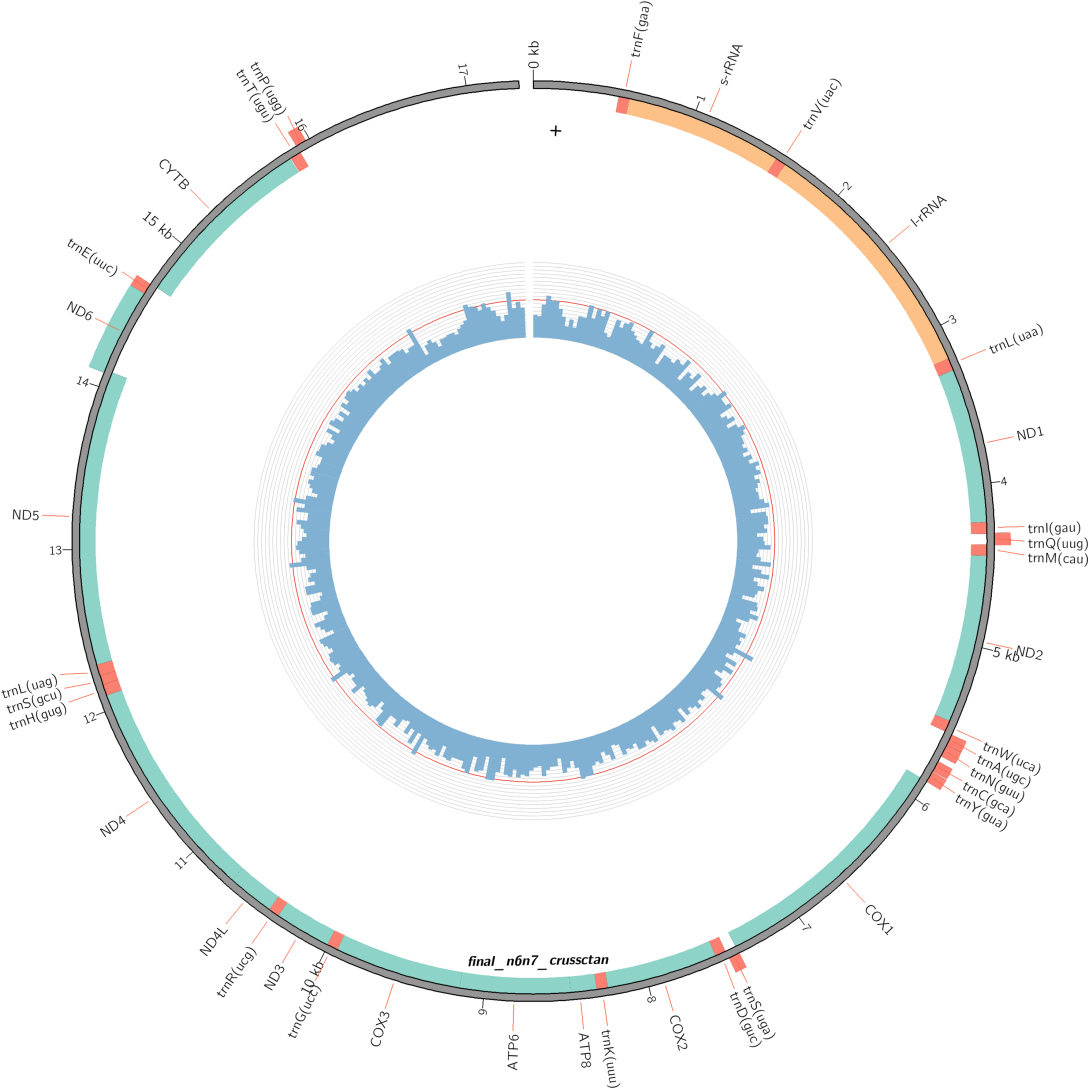
Circle plot visualization of the genome annotation. Inner blue ring depicts the read coverage. Numbers refer to kilobase pair positions. Green, red and yellow colors show position of protein coding, tRNA and rRNA genes, respectively.

The mitogenome annotation resulted in finding all 37 genes expected in animal mitogenomes, including 13 protein coding, 22 tRNA and 2 rRNA genes (Figure 4). No stop codon was seen in the coding regions of the protein‐coding gene. This new mitogenome sequence and annotation were deposited in GenBank (GenBank accession number: OR951920).

The authenticity of our DNA (the mapped reads) was supported by clear sign of C‐T deamination damage at the 5′ termini, observed by 30% increase in C to T transitions at that position (Table S3; Figure S1).

**FIGURE 5:**
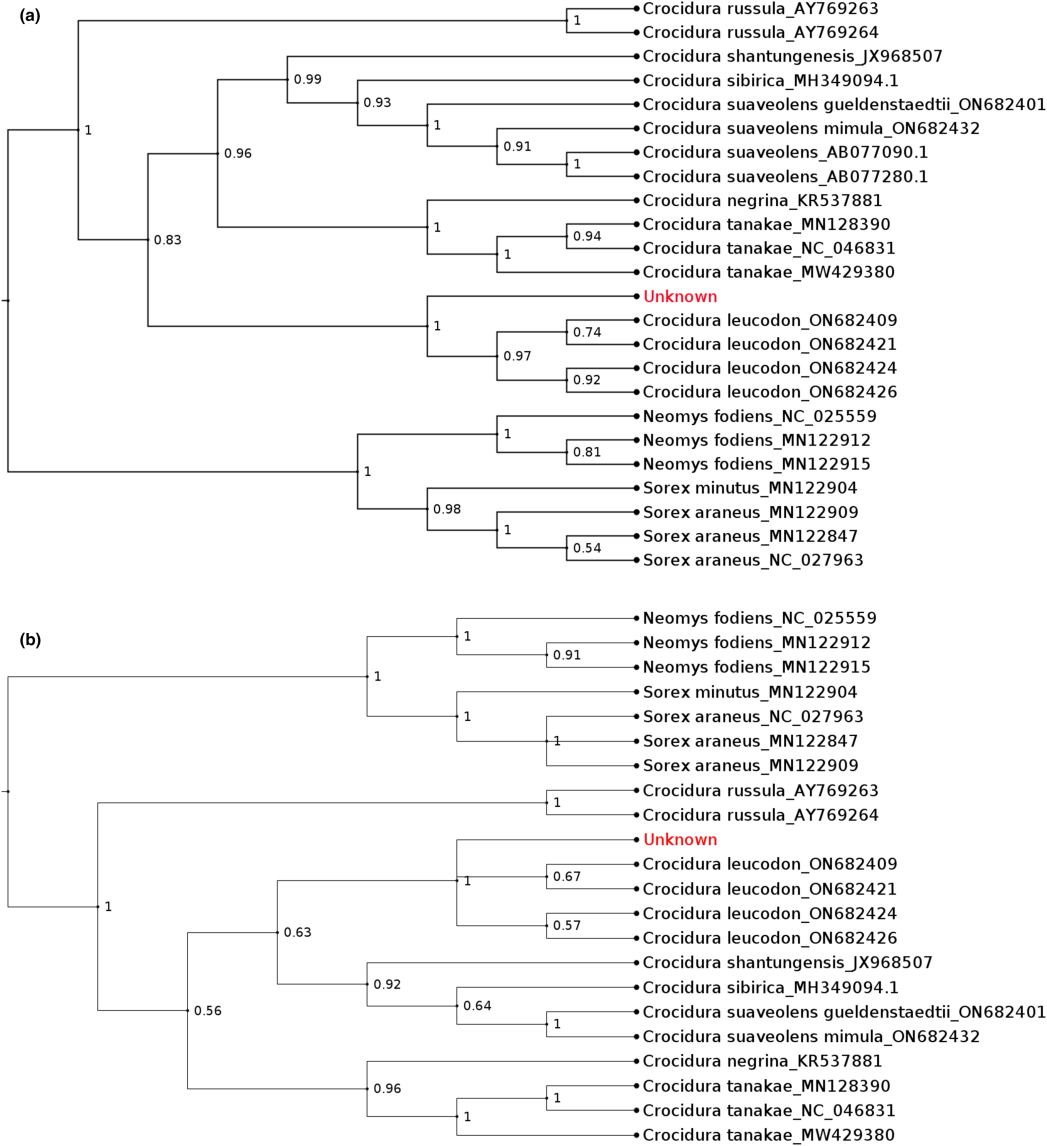
Bayesian phylogenetic tree derived from *Cytb* (a) and *COI* (b) dataset. Branch numbers refer to the Bayesian posterior probability values. The trees were generated using MrBayes 3.2.6 and visualized in FigTree v1.4.4 rooted via midpoint rooting. Our ancient shrew sample is here denoted “Unknown”.

Our phylogenies estimated by MrBayes and IQ‐Tree were highly congruent for both *Cytb* and *COI* genes; the three genera (i.e., *Crocidura, Neomys* and *Sorex*) and all the species were segregated as expected. Therefore, only Bayesian Inference (BI) gene trees are shown here, whereas maximum likelihood (ML) trees are included in the Figure S2. Both analyses confidently (i.e., with Bayesian posterior probability of 1.0 for both genes, ML bootstrap of 99 (*Cytb*) and 100 (*COI*)) placed the ancient shrew sample together with *C. leucodon* (Figure 5) but on a separate branch than the previously published *C. leucodon* sequences.

While the pairwise identity in alignment matrix (as calculated in Geneious) among the modern *C. leucodon* individuals was very high, ranging from 99.4% to 99.8% in *COI* and 99.1% to 99.8% in *cytB*, the ancient shrew proved slightly differentiated from the modern ones with pairwise identities ranging from 96.9% to 97.2% (*COI*) and 96.5% to 96.8% (*cytB*).

## 4 DISCUSSION

The unexpected identification of a bicolored shrew (*C. leucodon*) from the Late Bronze Age in Denmark not only highlights a remarkable zoological discovery but also raises questions about the historical distribution of this species in continental Scandinavia. While a skull and mandible from a modern white‐toothed shrew (*Crocidura sp*) was discovered in 2004 through the study of tens of thousands of micromammalian bones from barn owl pellets (Laursen, 2024), our ancient DNA analysis is the first to confirm the presence of *C. leucodon* in Denmark, and the first recorded presence of any member of the genus in this region in prehistoric times.

**FIGURE 6:**
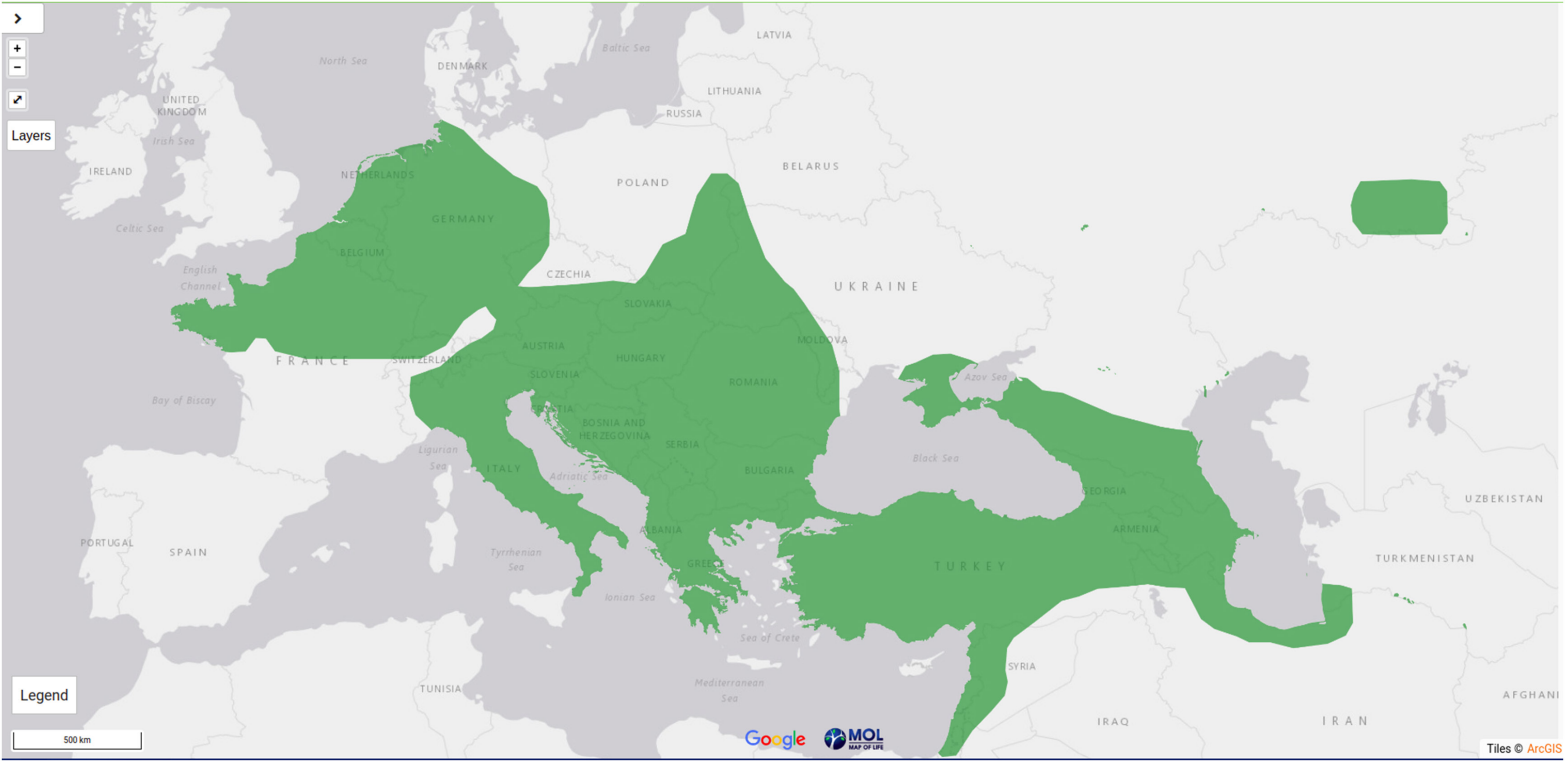
Current distribution map of *Crocidura leucodon*, as shown in Map of Life (MOL), accessed December 2023 at https://mol.org/species/.

Today *C. leucodon* occurs in Europe and western Asia from France to the Caspian Sea (Shenbrot et al., 2021) (Figure 6). At its northern range, *C. leucodon* is broadly confined by the Kiel Canal (Nord‐Ostsee‐Kanal) in Germany (Borkenhagen, 1993; Shenbrot et al., 2021), and is synanthropic, preferring artificial landscapes (gardens/fields) and houses feeding on invertebrates. It is, however, capable of living in a range of habitats from moist regions characterized by dense plant cover to damp environments in the mountain and open agricultural landscapes (Shenbrot et al., 2021; Vohralík et al., 2007).

According to the *Crocidura* records available in IANUS (Heinrich et al., 2016), a database of published zooarchaeological data from more than 8000 European archeological sites dated from the Late Pleistocene to Early Modern Times (Heinrich et al., 2016), species of *Crocidura* have been reported in Belgium, Switzerland, the Czech Republic, Germany, Spain, France, Great Britain, Greece, Hungary, Italy, Moldova, the Netherlands, Portugal, and Ukraine. This is while *C. leucodon* was only recorded in Germany, France, Greece, Hungary, and Ukraine. Similarly, *C. leucodon* was neither reported in the comprehensive overview of prehistoric species published by Aaris‐Sørensen (2009) nor in the GBIF data base (https://arter.dk/landing‐page) for Denmark.

The discovery of an archeological specimen of *C. leucodon* in a region where it no longer exists today triggers a debate on potential factors contributing to such occurrences. One likely scenario is that it may have been part of a fauna that existed during periods of different climatic conditions. Past climate changes, such as fluctuations in temperature and precipitation significantly influenced the distribution and composition of ecosystems (Svenning et al., 2015). Shrews, being highly sensitive to environmental conditions (Dokulilová et al., 2023), might have adjusted their ranges for example in response to climate change (Moritz & Agudo, 2013). We note that the transition between the Bronze Age and Iron Age in Northern Europe (at c. 800 bc) was marked by a shift from a relatively warm and dry to a wetter and colder climate (Barber et al., 2004; Bond et al., 1997; van Geel et al., 1996) which may have affected the *C. leucodon* distribution.

The phylogenetic placement of the ancient sample representing a basal split in the clade could be an effect of its age (genetic separation in time) or it could represent a distinct and now extinct local population that may have thrived in the region during the Late Bronze Age era. However, we note that higher sampling density across a wider range of the current distribution would be needed to clarify that.

While the bicolored shrew may have been more common in Bronze Age Denmark its remains are rarely found. This scarcity could be attributed to the selective preservation of certain bones over others or biased fossil deposition (Allentoft et al., 2010; Kattel et al., 2007), or the lack of fine‐meshed screening of sediments, and lack of scholarly attention, as well as spatial variability in the existence of paleontological sites (Polly, 2019) which might contribute to an assemblage that does not accurately represent the true composition of the local fauna. However, this observation of a single archeological specimen of a bicolored shrew in Denmark also calls for alternative hypotheses. One such explanation could be rare long‐range dispersal events, such as those reported in larger mammals (Moll et al., 2021). However, the mean dispersal progress of *C. russula* and *C. leucodon* in northern Germany was recorded about 2.5 km per year (Frank, 1984), and in Switzerland, the mean progress recorded is even smaller, at about 1 km per year (Vogel et al., 2002). The estimated distance between the Kiel Canal in Germany (i.e., the most northern distribution of the species), and where the species remains were found in Aalborg, Denmark is about 370 km. Based on these facts, it seems highly unlikely that long range migration is an explanation.

Moreover, human‐ or animal‐mediated movement of species or their remains introduces the possibility of intentional or unintentional transportation (Bullock et al., 2018; Poulakakis et al., 2005), potentially distorting the spatial and temporal context of the bone discovery. While we cannot completely rule out that transportation scenario, we find it improbable that the species was eaten by a migrating bird or carnivorous mammal somewhere south of Denmark before ending up near Aalborg. We are confident that our finding could not have been from a barn owl pellet, as barn owl is a late immigrant and was not present in Denmark before 1830s (Laursen, 2006). In light of the discussion above, we favor the hypothesis that the bicolored shrew was of local origin and thus part of the local fauna. The shift toward a wetter and colder climate in Denmark after the Bronze Age (Barber et al., 2004, Bond et al., 1997, van Geel et al., 1996) may explain why we do not have any finds of this species after that period.

The finding of a shrew bone dating to the Late Bronze Age in an excavated village from the Early Iron Age raises intriguing questions about this temporal discrepancy. Several factors could have contributed to this. First, there might have been a continuous habitation or reuse of the site over an extended period (Attema et al., 2019), leading to a mixing of sediments and remains from different time periods. Alternatively, geological or anthropogenic processes, such as soil erosion or construction/agricultural activities (Rosentau et al., 2013), could have displaced the shrew remains from its original context. Of relevance, it must be noted that 600–900 m south of the Iron Age settlement, several pits and longhouses were excavated in an earlier campaign. Most of these features were dated to the Bronze Age according to ^14^C dates and finds of ceramics (Internal report, Danish national database of archeological surveys). It is likely that the fields surrounding this Bronze Age settlement include the area that later was settled in the Iron Age, and activity in the latter may have resulted in displacement of the shrew remains.

In conclusion, the discovery of a *C. leucodon* bone from the Late Bronze Age in Denmark offers a novel perspective on prehistorical biodiversity and environmental dynamics in Scandinavia. As discussed earlier, the complexity of interpreting ancient bone assemblages emphasizes the necessity for a comprehensive approach when reconstructing the ecological history of a region. Exploring the prehistoric distribution and migration patterns of bicolored shrews in response to past environmental changes could provide valuable insights into their resilience and adaptive strategies of small mammals, particularly relevant in the context of contemporary climate change. Lastly, the mitochondrial genome reconstructed in this study serves as a valuable resource for future research, facilitating investigations into phylogenetic and population genetics in both extinct and extant shrew species.

Lastly, given the importance of the first DNA‐confirmed record of *C. leucodon* in Denmark, we suggest updating the records available on databases such as GBIF (https://arter.dk/landing‐page) accordingly.

## AUTHOR CONTRIBUTIONS

**Mahsa Mousavi‐Derazmahalleh:** Formal analysis (lead); writing – original draft (equal); writing – review and editing (equal). **Niels Haue:** Investigation (equal); writing – review and editing (equal). **Marie Kanstrup:** Formal analysis (supporting); investigation (equal); writing – review and editing (equal). **Jørgen T. Laursen:** Formal analysis (supporting); investigation (equal); writing – review and editing (equal). **Sherralee S. Lukehurst:** Formal analysis (supporting); writing – review and editing (equal). **Jacob Kveiborg:** Conceptualization (equal); formal analysis (supporting); investigation (equal); writing – review and editing (equal). **Morten E. Allentoft:** Conceptualization (equal); formal analysis (supporting); investigation (equal); writing – original draft (equal); writing – review and editing (equal).

## CONFLICT OF INTEREST STATEMENT

There are no conflicts of interest.

## Supporting information


Data S1.

## Data Availability

The mitochondrial genome is available on GenBank under accession number OR951920. The sequencing data can be accessed in Dryad data repository (https://doi.org/10.5061/dryad.3r2280gpn).

## References

[ece311680-bib-0001] Aaris‐Sørensen, K. (2009). Diversity and dynamics of the mammalian fauna in Denmark throughout the last glacial–interglacial cycle, 115–0 kyr BP. Fossils and Strata, 57, 1–59.

[ece311680-bib-0002] Allentoft, M. E. , Bunce, M. , Scofield, R. P. , Hale, M. L. , & Holdaway, R. N. (2010). Highly skewed sex ratios and biased fossil deposition of moa: Ancient DNA provides new insight on New Zealand's extinct megafauna. Quaternary Science Reviews, 29, 753–762.

[ece311680-bib-0003] Allentoft, M. E. , Sikora, M. , Sjögren, K.‐G. , Rasmussen, S. , Rasmussen, M. , Stenderup, J. , Damgaard, P. B. , Schroeder, H. , Ahlström, T. , Vinner, L. , & Malaspinas, A. S. (2015). Population genomics of bronze age Eurasia. Nature, 522, 167–172. 26062507 10.1038/nature14507

[ece311680-bib-0004] Andrews, S. (2010). FastQC: A quality control tool for high throughput sequence data. Babraham Bioinformatics, Babraham Institute.

[ece311680-bib-0005] ARTER.DK . (2023). *Arter* [Online]. https://arter.dk/landing‐page

[ece311680-bib-0006] Attema, P. , Jacobsen, J. K. , Colelli, C. , Ippolito, F. , Mittica, G. P. , & Saxkjaer, S. G. (2019). The bronze and iron Age habitation on Timpone della Motta in the light of recent research. Analecta Romana Instituti Danici, 43, 25–90.

[ece311680-bib-0007] Barber, K. E. , Chambers, F. M. , & Maddy, D. (2004). Late Holocene climatic history of northern Germany and Denmark: Peat macrofossil investigations at Dosenmoor, Schleswig‐Holstein, and Svanemose, Jutland. Boreas, 33, 132–144.

[ece311680-bib-0008] Bond, G. , Showers, W. , Cheseby, M. , Lotti, R. , Almasi, P. , Demenocal, P. , Priore, P. , Cullen, H. , Hajdas, I. , & Bonani, G. (1997). A pervasive millennial‐scale cycle in North Atlantic Holocene and glacial climates. Science, 278, 1257–1266.

[ece311680-bib-0009] Borkenhagen, P. (1993). Atlas der Säugetiere Schleswig‐Holsteins. Landesamt für Naturschutz Und Landschaftspflege Schleswig‐Holstein.

[ece311680-bib-0010] Brace, S. , Thomas, J. A. , Dalén, L. , Burger, J. , Macphee, R. D. , Barnes, I. , & Turvey, S. T. (2016). Evolutionary history of the Nesophontidae, the last unplaced recent mammal family. Molecular Biology and Evolution, 33, 3095–3103. 27624716 10.1093/molbev/msw186

[ece311680-bib-0011] Brown, T. A. , Nelson, D. E. , Vogel, J. S. , & Southon, J. R. (1988). Improved collagen extraction by modified Longin method. Radiocarbon, 30, 171–177.

[ece311680-bib-0012] Bullock, J. M. , Bonte, D. , Pufal, G. , da Silva Carvalho, C. , Chapman, D. S. , García, C. , García, D. , Matthysen, E. , & Delgado, M. M. (2018). Human‐mediated dispersal and the rewiring of spatial networks. Trends in Ecology & Evolution, 33, 958–970. 30314915 10.1016/j.tree.2018.09.008

[ece311680-bib-0013] Churchfield, S. , & Rychlik, L. (2006). Diets and coexistence in Neomys and Sorex shrews in Białowieża forest, eastern Poland. Journal of Zoology, 269, 381–390.

[ece311680-bib-0014] Darriba, D. , Taboada, G. L. , Doallo, R. , & Posada, D. (2012). jModelTest 2: More models, new heuristics and parallel computing. Nature Methods, 9, 772. 10.1038/nmeth.2109 PMC4594756 22847109

[ece311680-bib-0015] Dokulilová, M. , KROJEROVÁ‐Prokešová, J. , Heroldová, M. , Čepelka, L. , & Suchomel, J. (2023). Population dynamics of the common shrew (Sorex araneus) in central European forest clearings. European Journal of Wildlife Research, 69, 1–16.

[ece311680-bib-0016] Frank, F. (1984). Zur Arealverschiebung zwischen Crocidura russula und *C. leucodon* in NW‐Deutschland und zum wechselseitigen Verhältnis beider Arten. Zeitschrift für Säugetierkunde, 49, 65–70.

[ece311680-bib-0017] Hahn, C. , Bachmann, L. , & Chevreux, B. (2013). Reconstructing mitochondrial genomes directly from genomic next‐generation sequencing reads—A baiting and iterative mapping approach. Nucleic Acids Research, 41, e129. 23661685 10.1093/nar/gkt371 PMC3711436

[ece311680-bib-0018] Heinrich, D. , von den Driesch, A. , & Benecke, N. (2016). Holozängeschichte der Tierwelt Europas. Datensammlung Hrsg. v. IANUS.

[ece311680-bib-0019] Hope, A. G. , Waltari, E. , Morse, N. R. , Flamme, M. , Cook, J. A. , & Talbot, S. L. (2017). Small mammals as indicators of climate, biodiversity, and ecosystem change. Alaska Park Science, 16, 72–78.

[ece311680-bib-0020] Huelsenbeck, J. P. , & Ronquist, F. (2001). MRBAYES: Bayesian inference of phylogenetic trees. Bioinformatics, 17, 754–755. 11524383 10.1093/bioinformatics/17.8.754

[ece311680-bib-0021] Hutterer, R. (2005). Homology of unicuspids and tooth nomenclature in shrews. Advances in the Biology of Shrews, 397–404.

[ece311680-bib-0022] Jónsson, H. , Ginolhac, A. , Schubert, M. , Johnson, P. L. , & Orlando, L. (2013). mapDamage2. 0: Fast approximate Bayesian estimates of ancient DNA damage parameters. Bioinformatics, 29, 1682–1684. 23613487 10.1093/bioinformatics/btt193 PMC3694634

[ece311680-bib-0023] Jørkov, M. L. S. , Heinemeier, J. , & Lynnerup, N. (2006). Evaluating bone collagen extraction methods for stable isotope analysis in dietary studies. Journal of Archaeological Science, 34, 1824–1829.

[ece311680-bib-0024] Kattel, G. , Battarbee, R. , Mackay, A. , & Birks, H. (2007). Are cladoceran fossils in lake sediment samples a biased reflection of the communities from which they are derived? Journal of Paleolimnology, 38, 157–181.

[ece311680-bib-0025] Kristiansen, S. M. , Ljungberg, T. E. , Christiansen, T. T. , Dalsgaard, K. , Haue, N. , Greve, M. H. , & Nielsen, B. H. (2021). Meadow, marsh and lagoon: Late Holocene coastal changes and human–environment interactions in northern Denmark. Boreas, 50, 279–293.

[ece311680-bib-0026] Krystufek, B. , Davison, A. , & Griffiths, H. (2000). Evolutionary biogeography of water shrews (Neomys spp.) in the western Palaearctic region. Canadian Journal of Zoology, 78, 1616–1625.

[ece311680-bib-0027] Kveiborg, J. , & Olsen, J. (2023). Targeted radiocarbon dating of animal bones from ritual wetland depositions in early iron Age. Journal of Archaeological Science: Reports, 47, 103766.

[ece311680-bib-0028] Laursen, J. T. (2006). Danmarks Ugler. Apollo Books.

[ece311680-bib-0029] Laursen, J. T. (2024). Status, bestandsudvikling og fødevalg for Danmarks ynglende ugler ca. 1960–2021 (with English summary: Status, population trends and food selection in Denmark's breeding owls 1960–2021). Dansk Ornitologisk Forenings Tidsskrift, 118, 65–79.

[ece311680-bib-0030] Li, H. , & Durbin, R. (2009). Fast and accurate short read alignment with burrows–wheeler transform. Bioinformatics, 25, 1754–1760. 19451168 10.1093/bioinformatics/btp324 PMC2705234

[ece311680-bib-0031] Li, H. , Handsaker, B. , Wysoker, A. , Fennell, T. , Ruan, J. , Homer, N. , Marth, G. , Abecasis, G. , Durbin, R. , & 1000 Genome Project Data Processing Subgroup . (2009). The sequence alignment/map format and SAMtools. Bioinformatics, 25, 2078–2079. 19505943 10.1093/bioinformatics/btp352 PMC2723002

[ece311680-bib-0032] Li, H. , Li, Y. , Motokawa, M. , Wu, Y. , Harada, M. , & Li, Y. (2023). The effectiveness of molecular, karyotype and morphological methods in the identification of morphologically conservative sibling species: An integrative taxonomic case of the Crocidura attenuata species complex in mainland China. Animals, 13, 643. 36830430 10.3390/ani13040643 PMC9951653

[ece311680-bib-0033] Longin, R. (1971). New method of collagen extraction for radiocarbon dating. Nature, 230, 241–242. 4926713 10.1038/230241a0

[ece311680-bib-0034] Mackiewicz, P. , Moska, M. , Wierzbicki, H. , Gagat, P. , & Mackiewicz, D. (2017). Evolutionary history and phylogeographic relationships of shrews from Sorex araneus group. PLoS One, 12, e0179760. 28650986 10.1371/journal.pone.0179760 PMC5484494

[ece311680-bib-0035] Meng, G. , Li, Y. , Yang, C. , & Liu, S. (2019). MitoZ: A toolkit for animal mitochondrial genome assembly, annotation and visualization. Nucleic Acids Research, 47, e63. 30864657 10.1093/nar/gkz173 PMC6582343

[ece311680-bib-0036] Meyer, M. , & Kircher, M. (2010). Illumina sequencing library preparation for highly multiplexed target capture and sequencing. Cold Spring Harbor Protocols, 2010, t5448. 10.1101/pdb.prot5448 20516186

[ece311680-bib-0037] Minh, B. Q. , Schmidt, H. A. , Chernomor, O. , Schrempf, D. , Woodhams, M. D. , VON Haeseler, A. , & Lanfear, R. (2020). IQ‐TREE 2: New models and efficient methods for phylogenetic inference in the genomic era. Molecular Biology and Evolution, 37, 1530–1534. 32011700 10.1093/molbev/msaa015 PMC7182206

[ece311680-bib-0038] Moll, R. J. , Mcroberts, J. T. , Millspaugh, J. J. , Wiskirchen, K. H. , Sumners, J. A. , Isabelle, J. L. , Keller, B. J. , & Montgomery, R. A. (2021). A rare 300 kilometer dispersal by an adult male white‐tailed deer. Ecology and Evolution, 11, 3685–3695. 33976768 10.1002/ece3.7354 PMC8093661

[ece311680-bib-0039] Moritz, C. , & Agudo, R. (2013). The future of species under climate change: Resilience or decline? Science, 341, 504–508. 23908228 10.1126/science.1237190

[ece311680-bib-0040] Mukhacheva, S. , & Tolkachev, O. (2022). Long‐distance dispersal of two species of shrews (Sorex caecutiens Laxmann, 1788 and Sorex minutus Linnaeus, 1766). Mammalia, 86, 591–595.

[ece311680-bib-0041] Neves, T. , BORDA‐DE‐Água, L. , Mathias, M. D. L. , & Tapisso, J. T. (2021). The influence of the interaction between climate and competition on the distributional limits of European shrews. Animals, 12, 57. 35011163 10.3390/ani12010057 PMC8749581

[ece311680-bib-0042] Neves, T. , Tapisso, J. T. , Porto, M. , Pereira, H. M. , Mathias, M. L. , & Borda‐De‐Água, L . (2019). The role of competition in driving species global distributions: Soricid shrews as a case study. Journal of Biogeography, 46, 134–144.

[ece311680-bib-0043] Niethammer, J. , & Krapp, F. (1990). vol 3/1.: Insektenfresser—Herrentiere. In Handbuch der Säugetiere Europas. Akademische Verlagsgesellschaft.

[ece311680-bib-0044] Olsen, J. , Tikhomirov, D. , Grosen, C. , Heinemeier, J. , & Klein, M. (2017). Radiocarbon analysis on the new AARAMS 1MV Tandetron. Radiocarbon, 59, 905–913.

[ece311680-bib-0045] Orlando, L. , Ginolhac, A. , Zhang, G. , Froese, D. , Albrechtsen, A. , Stiller, M. , Schubert, M. , Cappellini, E. , Petersen, B. , Moltke, I. , & Johnson, P. L. (2013). Recalibrating Equus evolution using the genome sequence of an early middle Pleistocene horse. Nature, 499, 74–78. 23803765 10.1038/nature12323

[ece311680-bib-0046] Polly, P. D. (2019). Climate, diversification and refugia in the common shrew: Evidence from the fossil record. In Shrews, chromosomes and speciation. Cambridge University Press Cambridge.

[ece311680-bib-0047] Poulakakis, N. , Lymberakis, P. , Paragamian, K. , & Mylonas, M. (2005). Isolation and amplification of shrew DNA from barn owl pellets. Biological Journal of the Linnean Society, 85, 331–340.

[ece311680-bib-0048] Rambaut, A. (2006). FigTree [Online].

[ece311680-bib-0049] Ramsey, C. B. (2009). Bayesian analysis of radiocarbon dates. Radiocarbon, 51, 337–360.

[ece311680-bib-0050] Reimer, P. J. , Austin, W. E. N. , Bard, E. , Bayliss, A. , Blackwell, P. G. , Bronk Ramsey, C. , Butzin, M. , Cheng, H. , Edwards, R. L. , Friedrich, M. , Grootes, P. M. , Guilderson, T. P. , Hajdas, I. , Heaton, T. J. , Hogg, A. G. , Hughen, K. A. , Kromer, B. , Manning, S. W. , Muscheler, R. , … Talamo, S. (2020). The IntCal20 northern hemisphere radiocarbon age calibration curve (0–55 cal kBP). Radiocarbon, 62, 725–757.

[ece311680-bib-0051] Richter, H. (1964). Bestimmung der Unterkiefer (Mandibulae) von *Crocidura* r. russula (Hermann, 1780) und *Crocidur*a l. *leucodon* (Hermann, 1780). Zeitschrift für Säugetierkunde, 29, 253.

[ece311680-bib-0052] Rofes, J. , & Cuenca‐Bescos, G. (2011). Evolutionary history and biogeography of the genus *Crocidura* (Mammalia, Soricidae) in Europe, with emphasis on *Crocidura kornfeldi* . Mammalian Biology, 76, 64–78.

[ece311680-bib-0053] Rohland, N. , & Hofreiter, M. (2007). Ancient DNA extraction from bones and teeth. Nature Protocols, 2, 1756–1762. 17641642 10.1038/nprot.2007.247

[ece311680-bib-0054] Rosentau, A. , Muru, M. , Kriiska, A. , Subetto, D. A. , Vassiljev, J. , Hang, T. , Gerasimov, D. , Nordqvist, K. , Ludikova, A. , Lõugas, L. , & Raig, H. (2013). Stoneage settlement and Holocene shore displacement in the Narva‐Luga Klint Bay area, eastern gulf of Finland. Boreas, 42, 912–931.

[ece311680-bib-0055] Rowe, R. J. , & Terry, R. C. (2014). Small mammal responses to environmental change: Integrating past and present dynamics. Journal of Mammalogy, 95, 1157–1174.

[ece311680-bib-0056] Santos, G. M. , Southon, J. R. , Griffin, S. , Beaupre, S. R. , & Druffel, E. R. M. (2007). Ultra small‐mass AMS ^14^C sample preparation and analyses at Kccams, UCI Facility. Nuclear Instruments and Methods in Physics Research Section B: Beam Interactions with Materials and Atoms, 259, 293–302.

[ece311680-bib-0057] Schubert, M. , Lindgreen, S. , & Orlando, L. (2016). AdapterRemoval v2: Rapid adapter trimming, identification, and read merging. BMC Research Notes, 9, 1–7. 26868221 10.1186/s13104-016-1900-2 PMC4751634

[ece311680-bib-0058] Shenbrot, G. , Hutterer, R. , Kryštufek, B. , Yigit, N. , Mitsainas, G. , & Palomo, L. (2021). Crocidura leucodon (amended version of 2016 assessment) [Online] .

[ece311680-bib-0059] Stuiver, M. , & Polach, H. A. (1977). Discussion of reporting ^14^C data. Radiocarbon, 19, 355–363.

[ece311680-bib-0060] Svenning, J.‐C. , Eiserhardt, W. L. , Normand, S. , Ordonez, A. , & Sandel, B. (2015). The influence of paleoclimate on present‐day patterns in biodiversity and ecosystems. Annual Review of Ecology, Evolution, and Systematics, 46, 551–572.

[ece311680-bib-0061] Van Geel, B. , Buurman, J. , & Waterbolk, H. T. (1996). Archaeological and palaeoecological indications of an abrupt climate change in The Netherlands, and evidence for climatological teleconnections around 2650 BP. Journal of Quaternary Science: Published for the Quaternary Research Association, 11, 451–460.

[ece311680-bib-0062] Vogel, J. S. , Southon, J. R. , Nelson, D. E. , & Brown, T. A. (1984). Performance of catalytically condensed carbon for use in accelerator mass spectrometry. Nuclear Instruments and Methods in Physics Research Section B: Beam Interactions with Materials and Atoms, 5, 289–293.

[ece311680-bib-0063] Vogel, P. , Jutzeler, S. , Rulence, B. , & Reutter, B. A. (2002). Range expansion of the greater white‐toothed shrew *Crocidura russula* in Switzerland results in local extinction of the bicoloured white‐toothed shrew *C. Leucodon* . Acta Theriologica, 47, 15–24.

[ece311680-bib-0064] Vogel, R. , Hutterer, R. , & Sara, M. (1989). The correct name, species diagnosis, and distribution of the Sicilian shrew. Bonner Zoologische Beiträge, 40, 243–248.

[ece311680-bib-0065] Vohralík, V. , Zima, J. , & Kryštufek, B. (2007). Crocidura leucodon (Europe assessment) [Online] .

[ece311680-bib-0066] Von Merten, S. , & Siemers, B. M. (2012). Exploratory behaviour in shrews: Fast‐lived Sorex versus slow‐lived *Crocidura* . Animal Behaviour, 84, 29–38.

[ece311680-bib-0067] Young, R. L. , Sweeney, M. J. , & Badyaev, A. V. (2010). Morphological diversity and ecological similarity: Versatility of muscular and skeletal morphologies enables ecological convergence in shrews. Functional Ecology, 24, 556–565.

