# Supplementary material for: Far away from home? Ancient DNA shows the presence of bicolored shrew (Crocidura leucodon) in Bronze Age Denmark
Source: Ecol Evol. 2024 Jul 2;14(7):e11680. doi: 10.1002/ece3.11680 (PMC11219242; doi:10.1002/ece3.11680)
Supplement: Supplementary file 1 — Data S1. [file ECE3-14-e11680-s001.docx]

**Supplementary Files**

**Far away from home? Ancient DNA shows presence of bicoloured shrew (*Crocidura leucodon*) in Bronze Age Denmark**

**Authors**

Mahsa Mousavi-Derazmahalleh^1^**^*^**, Niels Haue^2^, Marie Kanstrup^3^, Jørgen Terp Laursen^4^, Sherralee S. Lukehurst^1^, Jacob Kveiborg^5^, Morten E. Allentoft^1,6^**^*^**

**Table S1.** List of identified crania and mandibles of micromammals (insectivores and rodents) from the Iron Age site ÅHM 6023 Postgården

|  | **Number of identified specimens (NISP)** |
| --- | --- |
| **Insectivores** |  |
| Common shrew (*Sorex araneus*) | 1 |
| Bicolored white-toothed shrew (*Crosidura leucodon*) | 3 |
| **Muridae** |  |
| Eurasian harvest mouse (*Micromys minutus*) | 4 |
| Yellow-necked field mouse/Wood mouse (cf. *A.flavicollis/A. sylvaticus*)? | 1 |
| *Apodemus* sp. | 18 |
| House mouse (*Mus musculus)* | 5 |
| House mouse (cf. *Mus musculus)*? | 1 |
| **Cricetidae** |  |
| Bank vole (*Clethrionomys glareolus*) | 1 |
| Eurasian water vole (*Arvicola amphibius*) | 15 |
| Common vole *(Microtus arvalis*) | 3 |
| *Microtus* sp. | 6 |
| Northern birch mouse (cf. *Sicista betulina)?* | 1 |

**Table S2.** List of species sequences used for the phylogeny analyses. *Cytb* and *COI* Genes were extracted from these complete mitogenomes.

| **Species** | **GenBank accession number** |
| --- | --- |
| *Neomys fodiens* | NC_025559 (KM092492) |
| *Neomys fodiens* | MN122915 |
| *Neomys fodiens* | MN122912 |
| *Sorex araneus* | MN122909 |
| *Sorex araneus* | MN122847 |
| *Sorex araneus* | NC_027963 (KT210896) |
| *Sorex minutus* | MN122904 |
| *Crocidura russula* | AY769264 |
| *Crocidura russula* | AY769263 |
| *Crocidura shantungenesis* | JX968507 |
| *Crocidura sibirica* | MH349094.1 |
| *Crocidura negrina* | KR537881 |
| *Crocidura tanakae* | MN128390 |
| *Crocidura tanakae* | NC_046831 |
| *Crocidura tanakae* | MW429380 |
| *Crocidura leucodon* | ON682409 |
| *Crocidura leucodon* | ON682421 |
| *Crocidura leucodon* | ON682424 |
| *Crocidura leucodon* | ON682426 |
| *Crocidura suaveolens* | AB077280.1 |
| *Crocidura suaveolens* | AB077090.1 |
| *Crocidura suaveolens* | ON682401 |
| *Crocidura suaveolens* | ON682432 |

**Table S3.** Ancient DNA damage parameters estimated with mapDamage 2.0 (Jónsson et al., 2013). λ is the estimated fraction of bases positioned in single-stranded overhangs, and δs is the estimated C → T transition rate in the single-stranded overhangs.

| C to T frequency increase at 5'end pos 1 (%) | δS mean | δS 2.5% quantile | δS 97.5% quantile | λ mean | λ 2.5% quantile | λ 97.5% quantile |
| --- | --- | --- | --- | --- | --- | --- |
| 30 | 65 | 60 | 72 | 29 | 24 | 34 |


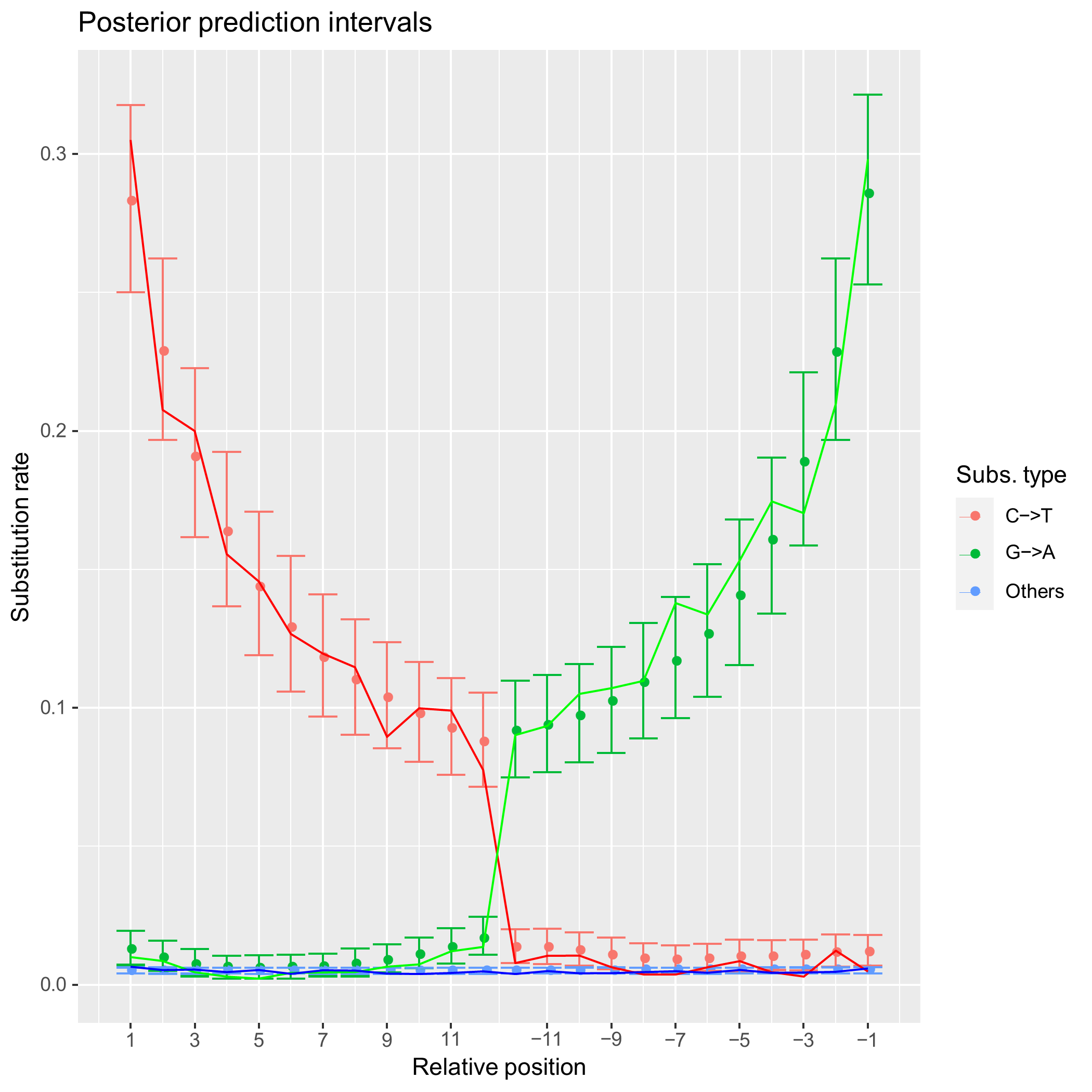


**Fig S1.** 95% Posterior predictive intervals for the substitution frequencies. The x-axis is the position from the 5’ end and y-axis is the substitution frequency.


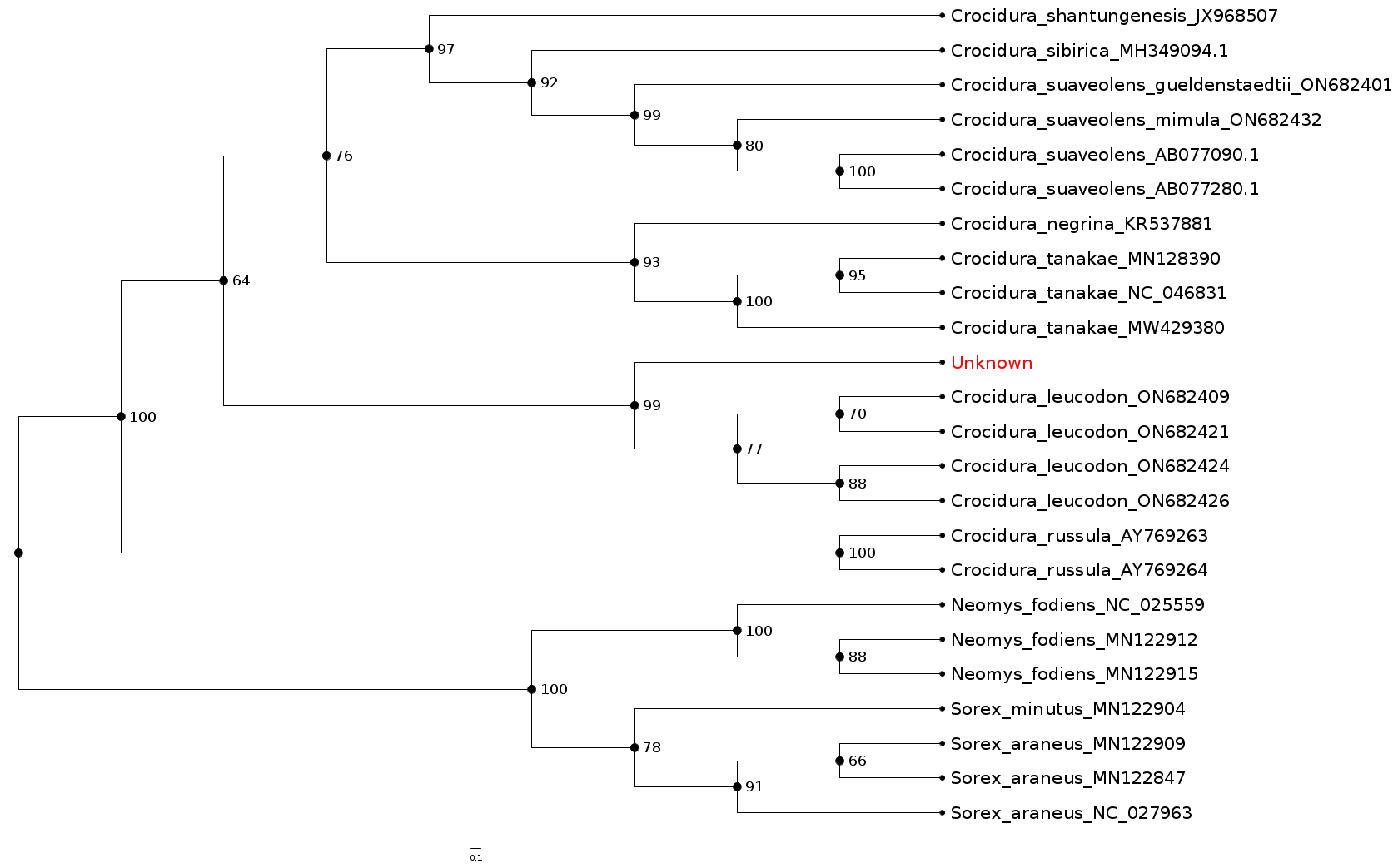


**(a)**

**
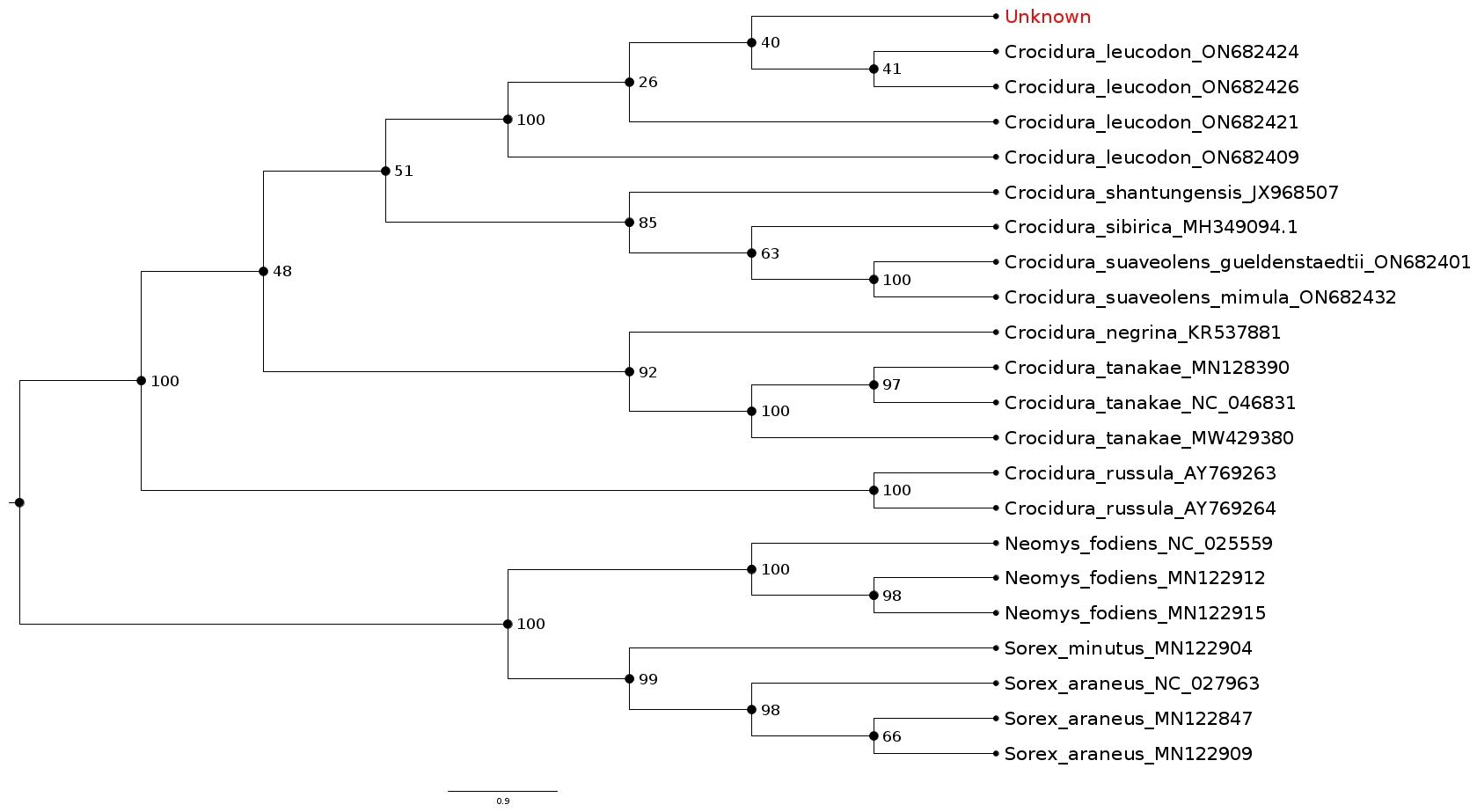
**

**(b)**

**Figure S2.** Maximum-Likelihood phylogenetic tree derived from *Cytb* **(a)** and *COI* **(b)** dataset. Branch numbers refer to bootstrap values. The trees were generated using IQ-Tree and visualized in Figtree.
